# Epithelial-mesenchymal reprogramming by KLF4-regulated Rictor expression contributes to metastasis of non-small cell lung cancer cells

**DOI:** 10.7150/ijbs.73548

**Published:** 2022-07-18

**Authors:** Huiling Zhou, Qing Guan, Xuyang Hou, Lijun Liu, Li Zhou, Wei Li, Haidan Liu

**Affiliations:** 1 Department of Cardiovascular Surgery, The Second Xiangya Hospital of Central South University, Changsha, Hunan, China.; 2 Clinical Center for Gene Diagnosis and Therapy, The Second Xiangya Hospital of Central South University, Changsha, Hunan, China.; 3 Department of Radiology, The Third Xiangya Hospital of Central South University, Changsha, Hunan, China.; 4 Department of Pathology, The Xiangya Hospital of Central South University, Changsha, Hunan, China.

**Keywords:** Rictor, KLF4, EMT, metastasis, NSCLC

## Abstract

Non-small cell lung cancer (NSCLC) is one of the deadliest cancers in the world. Metastasis is considered one of the leading causes of treatment failure and death in NSCLC patients. A crucial factor of promoting metastasis in epithelium-derived carcinoma has been considered as epithelial-mesenchymal transition (EMT). Rictor, one of the components of mTORC2, has been reportedly involved in EMT and metastasis of human malignancies. However, the regulatory mechanisms of Rictor, Rictor-mediated EMT and metastasis in cancers remain unknown. Our present study indicates that Rictor is highly expressed in human NSCLC cell lines and tissues and is regulated, at least partially, at the transcriptional level. Knockdown of Rictor expression causes phenotype alterations through EMT, which is accompanied by the impairment of migration and invasion ability in NSCLC cells. Additionally, we have cloned and identified the human *Rictor* core promoter for the first time and confirmed that transcription factor KLF4 directly binds to the *Rictor* promoter and transcriptionally upregulated Rictor expression. Knockdown of KLF4 results in Rictor's downregulation accompanied by a series of characteristic changes of mesenchymal-epithelial transition (MET) and significantly decreases migration, invasion as well as metastasis of NSCLC cells. Re-introducing Rictor in KLF4-knockdown NSCLC cells partially reverses the epithelial phenotype to the mesenchymal phenotype and attenuates the inhibition of cell migration and invasion caused by KLF4 knocking down. Knockdown of KLF4 prevents mTOR/Rictor interaction and metastasis of NSCLC* in vivo*. The understanding of the regulator upstream of Rictor may provide an opportunity for the development of new inhibitors and the rational design of combination regimens based on different metastasis-related molecular targets and finally prevents cancer metastasis.

## Introduction

Non-small cell lung cancer (NSCLC) is a primary cause of cancer death worldwide 1. The incidence is increasing, particularly in developing nations where the smoking rate and air pollution are increasing 1, 2. Although the early diagnosis has been improved and the standard treatment has been developed during the past few decades, NSCLC is often diagnosed at a later stage, and the overall 5-year survival rate remains less than 15%. Metastasis is a major cause of death in lung cancer patients 3. Hence, thoroughly understanding the biological process of metastasis is a prerequisite for developing novel strategies targeting metastasis.

Rapamycin-insensitive companion of mTOR (Rictor) is initially identified as a component of the mammalian target of rapamycin complex 2 (mTORC2) and is required for mTORC2 function 4. Rictor binding to mTOR, which forms mTORC2, modulates cell proliferation and survival in response to growth factors by promoting phosphorylation and activation of Akt/PKB, PKC, S6K and serum glucocorticoid-induced protein kinase (SGK) 5, 6 as well as modulates cytoskeletal dynamics 4, 7. In addition, Rictor reportedly exerts functions in an mTOR-independent manner. Rictor has been shown to directly interact with myosin Myo1c to form a Rictor/Myo1c complex which phosphorylates paxillin at Tyr118 and participates in cortical actin remodeling 8. Rictor/ILK interaction is also found, which can regulate Akt Ser473 phosphorylation and cell survival in the absence of mTOR expression 9. Rictor interacts with PKC ζ in the absence of mTOR and regulates cancer cell metastasis 10. Additionally, Rictor/Cullin-1 interaction functions as an E3 ligase, which promotes SGK1 ubiquitination and degradation 11. Thus, it is plausible that Rictor acts as a general scaffold protein and exerts different functions in an mTOR-independent manner. Rictor overexpression alone has been demonstrated to sufficiently induce malignant glioma formation in a transgenic mouse model, establishing Rictor as an oncoprotein 12. *Rictor* gene amplification 13-16 and Rictor protein overexpression 10, 17-21 have been found in various cancer types and correlated with tumor progression and metastasis, highlighting the potential therapeutic advantage for targeting Rictor. In addition, lung cancer reportedly appears to be one of the tumors with the highest frequency of *Rictor* amplification 13.

As an important reversible step, epithelial-mesenchymal transition (EMT) promotes carcinoma cells to dissociate from each other and migrate, invade and metastasize to distant organs 22. Thiery *et al*.^23^ proposed a reversible EMT model that carcinoma cells undergo EMT to invade and disseminate from the primary tumor; once arriving at distant sites, disseminated tumor cells need to undergo a reversion process termed mesenchymal-epithelial transition (MET) to form macrometastases. Owing to the clinical importance of this process, inhibition of EMT is an attractive therapeutic approach that can significantly alter disease outcomes. However, it remains unknown which pathways should be inhibited to reverse EMT. A recent report 24 demonstrated that TGFβ-1 induces Rictor expression, promotes Rictor/ILK complex formation, and regulates EMT in normal mouse epithelial NMe cells and human epithelial MCF10A cells. Gulhati *et al.* 21 reported that pharmacologic or genetic inhibition of Rictor induces a series of characteristic changes of MET and significantly decreases colorectal cancer cell migration, invasion and metastasis. These studies suggest that mesenchymal-epithelial reprogramming may be an important mechanism underlying the attenuated metastasis of cancer, supporting the targeting of Rictor as an anti-metastatic therapy. Although the function of Rictor in regulating growth and survival has been intensively researched, its special role in EMT, motility and metastasis of cancers is not well understood. Moreover, the regulatory mechanism of Rictor expression is yet fully unveiled. Therefore, understanding Rictor regulation is necessary for identifying its function in association with the EMT regulation.

KLF4, a zinc finger transcription factor of the Kruppel-like factor (KLF) family, binds to the relatively loose G/AG/AGGC/TGC/T sequence, which is present in the promoter of its target genes 25. By binding to the cis-element in the *p21* proximal promoter, KLF4 activates p21 expression, which causes cell-cycle arrest 26. KLF4 positively regulates u-PAR expression which facilitates the synthesis of u-PAR in colonic crypt luminal surface epithelial cells 27. KLF4 increases Bmp6 expression by directly binding to the *Bmp6* promoter. KLF4 can also directly upregulate the expression of EndMT (Endothelial-to-mesenchymal transition) markers, Fsp1 and Sca1, by binding to the *Fsp1* and *Sca1* promoters, respectively 28. KLF4 positively regulates flow-dependent Connexin40 (Cx40) expression by binding to three predicted KLF consensus binding sites in the *Cx40* promoter 29. KLF4 activates E-cadherin expression by binding the GC-boxes containing putative KLF4 sites of the *E-cadherin* promoter, resulting in alterations in epithelial cell morphology and migration 30. KLF4 binds the *RYBP* promoter to induce RYBP expression 31. As a transcriptional repressor, KLF4 downregulates Slug expression by binding to the *Slug* gene regulatory sequences, which regulates TGFβ-initiated prostatic EMT 32. KLF4 directly suppresses p53 expression by binding to the KLF4-responsive element in the* p53* promoter 33. Whether KLF4 functions as a transcriptional activator or repressor of target gene transcription by different mechanisms depends on the cell type or context.

Using NSCLC cells as models, the aim of the present study includes (1) Identification of cis-acting elements in human *Rictor* promoter; (2) Elucidation of key regulatory molecule that modulated Rictor expression; (3) Estimation effect of regulatory molecule on Rictor expression and its role in mesenchymal-epithelial reprogramming and metastasis of NSCLC. We have cloned the human *Rictor* core promoter and confirmed that transcription factor KLF4 directly binds to the *Rictor* promoter and transcriptionally upregulates Rictor expression. We found that epithelial-mesenchymal reprogramming by KLF4-regulated Rictor expression contributes to metastasis of NSCLC cells. Targeting the KLF4-Rictor axis may be a promising anti-tumor strategy to overcome metastasis in NSCLC.

## Materials and methods

### Reagents and antibodies

Chemical reagents for molecular biology and buffer preparation were purchased from Sigma-Aldrich (St. Louis, MO). Primary antibodies used for Western blot analysis included: KLF4 (#09-821) from Millipore; Rictor (#2114), KLF4 (#4038), Vimentin (#5741), Claudin-1 (#13255), β-catenin (#8480), Slug (#9585), E-cadherin (#3195) and p-Akt-Ser473 (#4060) from Cell Signaling Technology (Beverly, MA); Rictor (sc-81538) from Santa Cruz Biotechnology (Dallas, TX); Anti-HA tag (ab9110) from Abcam (Cambridge, UK); β-actin (A5316) from Sigma-Aldrich (St. Louis, MO). Secondary antibodies were anti-rabbit (sc-2004) and anti-mouse (sc-2005) IgG HRP were purchased from Santa Cruz Biotechnology (Dallas, TX).

### Clinical tissue sample collection

NSCLC tissues and the corresponding normal adjacent tissues were obtained from the Department of Thoracic Surgery, the Second Xiangya Hospital of Central South University with written informed consent (n = 22). Patients were diagnosed and classified by the Department of Pathology of the Second Xiangya Hospital following WHO guidelines. All the patients received no treatment before surgery.

### Cell lines and cell culture

Human NSCLC cells, including NCI-H23, NCI-H1299, NCI-H125, NCI-H358, NCI-H460, NCI-H1650 and HCC827 from American Type Culture Collection (ATCC, Manassas, VA) were cultured in medium (RPMI-1640) supplemented with 10% fetal bovine serum (FBS) and 1% antibiotics according to the ATCC protocols. A549 cells, immortalized human bronchial epithelial (HBE) cells and 293T cells were cultured as previously described 34. Before being frozen, cells were cytogenetically tested and authenticated. Frozen cells was thawed and maintained for 2 months (10 passages).

### Plasmid constructs

To generate wild-type, truncated, and site-directed mutated reporter vectors for the human *Rictor* promoter, PCR was used to amplify the* Rictor* promoter region from the human genomic DNA. We first amplified the DNA fragment containing the human *Rictor* promoter region spanning from -2000 to +96 bp around the transcription start site (TSS) of the *Rictor* gene, using a genomic DNA sample from normal HBE cells as templates. The product was cloned into* a pGL3-Basic* vector and designated as *pGL3-2000/+96*. To make serial truncated constructs of the *Rictor* promoter region, the* pGL3-2000/+96* vector was used as a template to amplify a series of the *Rictor* promoter truncated fragments (*-1101/+96*, *-820/+96*, *-413/+96*, *-201/+96*,* -144/+96*, *-101/+96*, *-37/+96*, *-11/+96*) and cloned into *pGL3-Basic* vector. The primers for cloning the constructs of human *Rictor* promoter were listed in **Supplementary Table 1**. The forward primer contained a restriction enzyme site of *Xho I* and the reverse primer contained a restriction enzyme site of *Hind III*. The potential KLF4 binding sites were predicted by programs (http://www.genomatix.de/cgi-bin, and http://www.generegulation.com/cgi-bin/pub/programs/alibaba2/webbaba2.cgi). Deletion mutant was created using a two-step PCR strategy where primers directed at nucleotides flanking the region to be deleted were used for amplification. Subsequent amplicons were digested and relegated to loop out the desired region. The deletion of KLF4-binding motif was designated as* pGL3-37/+96 Del KLF4*. The construction of the mutated KLF4 binding site was done by the generation of point mutation from the wild-type *Rictor* promoter region. Point mutations of the KLF4 binding site were also generated by a two-step PCR. The point mutant construct was designated as *pGL3-37/+96 Mut KLF4*. The primer sets used for generating mutations are listed in **Supplementary Table 2**. All of the constructs were confirmed by restriction mapping and DNA sequencing. Lentivirus plasmids containing *pLKO.1-shKLF4* (#1, TRCN0000005313; #2, TRCN0000005316) were purchased from Thermo Scientific. Lentivirus plasmids containing *pLKO.1-shRictor* (#1853 and #1854) 35, *pLKO.1-shGFP* (#30323), the lentiviral packaging plasmid *psPAX2* (#12260), the envelope plasmid* pMD2.G* (#12259) and the Rictor expression construct *myc-Rictor* (#1860) were obtained from Addgene (Watertown, MA). Transcription factors cDNA NFAT1(#11100), NFAT2 (#11101), cFos (#59140), ATF4 (#26114), p65 (#111190), p50 (#21965), Egr1 (#52724), cJun (#47443), JunB (#29687), HSF (#1948), SRF (#11977), Sp1 (#12096), Nrf2 (#21555), β-catenin (#16828), YAP1 (#18978), YAP2 (#19045), HIF1α (#18949), E2F1 (#24225), E2F2 (#24226), E2F4 (#10914), E2F5 (#24213), LEF1 (#27023), TCF4 (#16512), ELK-1 (#27156), Smad4 (#14959), KLF4 (#34593) were obtained from Addgene (Watertown, MA). Transcription factors cDNA C/EBPα, C/EBPβ, ATF5, cJun-cFos, USF1, HSF2, MZF1, ATF7 and Chop were kindly provided by Dr. Nak-Kyun Soung (Korea Research Institute of Bioscience and Biotechnology, Korea).

### Lentiviral infection and transient transfection

The generation of stable *Rictor* or *KLF4* knockdown NSCLC cell lines was performed as described previously 36. The knockdown of Rictor or KLF4 was confirmed by Western blot analysis (**Supplementary Figure 1A and 1B**). NSCLC cells were transiently transfected with the *pGL3-Basic* vector, the *pGL3-Rictor* promoter plasmid along with/without *pcDNA3.1-HA-KLF4 FL* (Addgene plasmid #34593) and Dual-Luciferase reporter assays were conducted as described previously 37. All experiments were performed in triplicate with three independent experiments.

### Real-time quantitative polymerase chain reaction (RT-qPCR)

RT-qPCR was performed as previously described 38. The primers for *Rictor*: Forward sequence: ggaagcctgttgatggtgat; Reverse sequence: ggcagcctgttttatggtgt. The primers for *GAPDH*: Forward sequence: tgttgccatcaatgacccctt; Reverse sequence: ctccacgacgtactcagcg.

### Cell viability assay

Cells (2×10^3^ per well) were seeded in 96-well plates and cell viability was examined at various timepoints using the WST-1 reagent (Roche, Mannheim, Germany) as described previously 38.

### Soft agar assay

The anchorage-independent growth of NSCLC cells (8×10^3^ per well) was assessed by soft agar assay according to previously described 38.

### Protein preparation and Western blot

Protein preparation and Western blot were carried out as reported previously 39.

### Co-immunoprecipitation (Co-IP) assay

Co-IP assays were performed as described previously 34. Antibodies were used for immunoprecipitation: Rictor (sc-81538, Santa Cruz Biotechnology), mTOR (#2972, Cell Signaling Technology), normal mouse IgG (NI03, Calbiochem), or normal rabbit IgG (NI01, Calbiochem). Immunocomplexes were resolved by SDS-PAGE and co-immunoprecipitated proteins were detected using mTOR (#2972) and Rictor (#2114) from Cell Signaling Technology (Beverly, MA), respectively.

### Chromatin immunoprecipitation (ChIP) assay

ChIP assays were done according to previously described 37. Antibodies were used for immunoprecipitation: KLF4 (sc-20691, Santa Cruz Biotechnology) and normal rabbit IgG (#NI01, Calbiochem). Primers designed to amplify the *Rictor* promoter regions present in the immunoprecipitated DNA are as follows: Forward sequence: gcgcggcgcgcggggaggggaaggggttc; Reverse sequence: ggagtgagggttgcagcgggcttacct. The amplified products (151 bp) were separated in 3% agarose gel containing ethidium bromide.

### Wound healing assay

The migration of NSCLC cells was investigated using a wound healing assay as described previously 34.

### Transwell invasion assay

Transwell invasion assay were carried out using chambers consisting 8.0-µm invasion inserts (Corning, USA) as described previously 34. Cells (1×10^5^ cells/well), suspended in FBS-free medium, were seeded on Matrigel (BD Bioscience)-coated top chamber. The lower chamber was filled with medium containing 20% FBS. After 24 h of incubation, non-invaded cells on the upper surface of the insert were wiped off by a cotton swab. The invaded cells on the underside of the insert were fixed with 4% formaldehyde and stained with 1% crystal violet. Five random fields were photographed and quantified.

### Immunohistochemical (IHC) Staining

The NSCLC tissues and the paired adjacent tissues were fixed, embedded, and subjected to IHC analysis as described previously 40. IHC staining were performed with antibodies against Rictor (1:100, ab70374, Abcam), KLF4 (1:100, sc-20691, Santa Cruz). Hematoxylin was used for counterstaining. The intensity was estimated by Image-Pro PLUS (v.6) and Image J (NIH) software programs.

### *In vivo* metastasis assay

All the experimentation for animals was approved by the Medical Research Animal Ethics Committee, Central South University, China. H1299 cell line was used for determining the knockdown of KLF4 on the establishment of metastatic tumors. Stable H1299-shGFP, H1299-shKLF4#1 or H1299-shKLF4#2 cells (2 × 10^6^) in 100 μL PBS buffer were injected into the lateral tail vein of 6-week-old female athymic nude mice in each group (n = 5). The mouse body weight was recorded. Six weeks after cell injection, the mice were euthanized. The lungs were removed and measured on a microbalance and then fixed in 10% formalin fixative for hematoxylin and eosin (H&E) staining. Lung tumor formation was observed and the number of lung tumors was counted 34.

### Statistical analysis

The statistical package SPSS (version 16.0 for Windows, SPSS Inc, Chicago, IL, USA) and GraphPad Prism (GraphPad 7.0, San Diego, CA, USA) were used for statistical analyses. All quantitative data were expressed as mean values ± S.D of three independent experiments. Differences between means were evaluated by Student's *t*-test or analysis of variance (ANOVA) when data were normally distributed. The statistical significance of the correlations between Rictor expression and clinicopathologic characteristics were assessed by χ^2^ test or Fisher's exact test. Pearson rank correlation was used for correlation tests. A probability value of *p* < 0.05 was used as the criterion for statistical significance.

## Results

### Rictor is highly expressed in human non-small cell lung cancer

**Figure 1:**
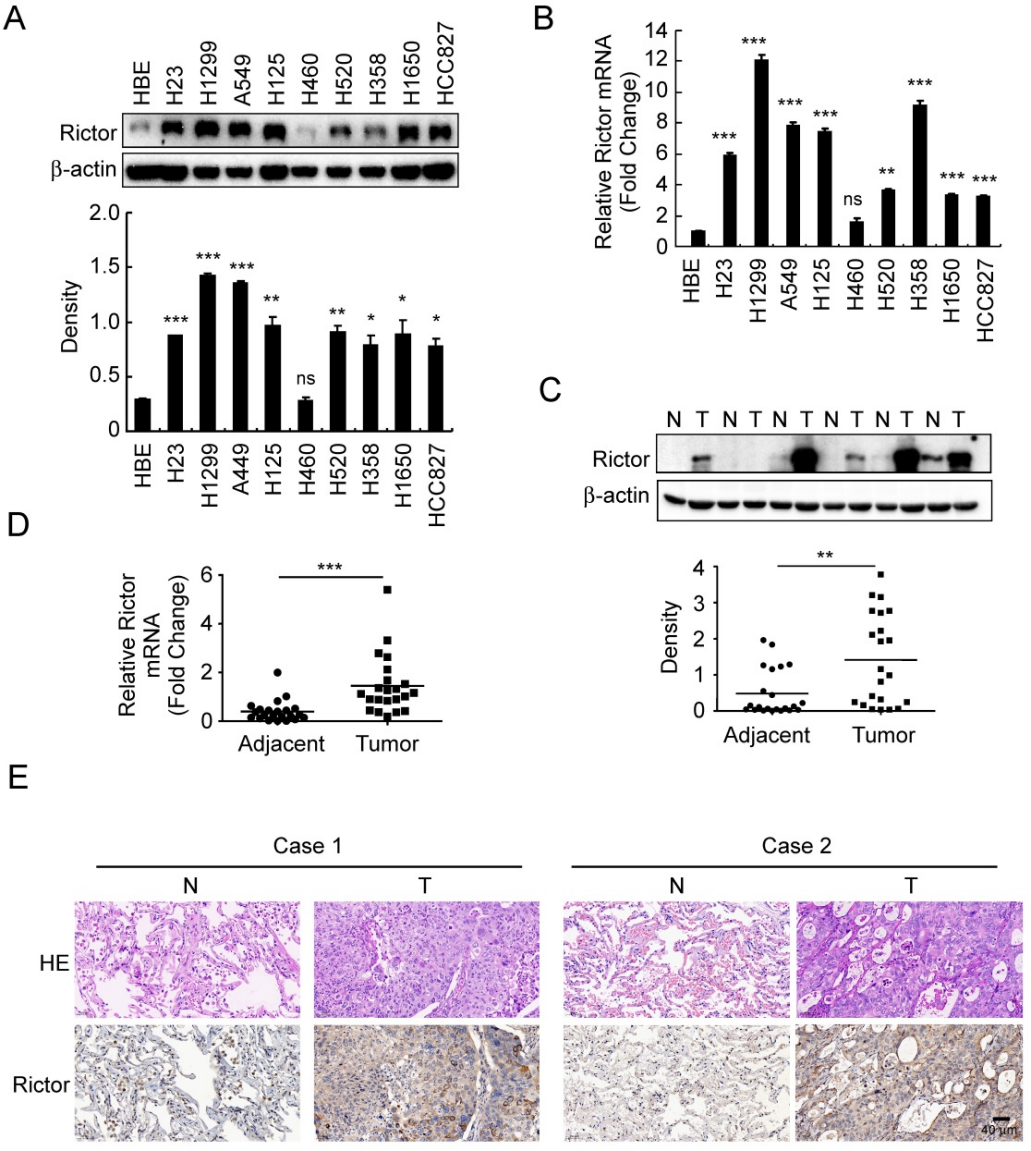
Expressions of Rictor in human non-small cell lung cancer. **A**, Western blot analysis was performed to examine Rictor expression in human NSCLC cell lines and immortalized HBE cells. β-actin was as a loading control (*upper panel*). The density of Rictor protein was shown (*lower panel*). *p<0.05; **p<0.01; ***p<0.001; ns, not statistically significant. **B**, expression of *Rictor* mRNA in human NSCLC cell lines and HBE cells was analyzed by RT-qPCR. **p<0.01; ***p<0.001; ns, not statistically significant. **C**, Rictor protein level in six representative NSCLC cases was assessed by Western blotting. β-actin was as a loading control. N, normal adjacent tissue; T, tumor (*upper panel*). Western blotting determined Rictor protein levels in the malignant and the corresponding normal adjacent tissues of 22 NSCLC patients* (lower panel)*. The intensity was evaluated using Image J (NIH) computer software. **p<0.01. **D**, expression of *Rictor* mRNA in the malignant and the corresponding normal adjacent tissues of 22 NSCLC patients was analyzed by RT-qPCR. ***p<0.001. **E**, representative figures of IHC staining for Rictor were detected on NSCLC tissues and the corresponding normal adjacent samples.

To investigate whether the expression of Rictor is related to human non-small cell lung cancer, Rictor protein level was examined by Western blotting in an immortalized human bronchial epithelial (HBE) cell line and nine human NSCLC cell lines. The result showed that except for the H460 cells, Rictor was highly expressed in almost all human NSCLC cell lines tested** (Figure 1A)**. The RT-qPCR results indicated that increased expression of *Rictor* mRNA levels in these NSCLC cell lines compared with that in HBE cells** (Figure 1B)**, which was in agreement with the result of the Western blotting analysis. We further determined Rictor levels in the NSCLC tissues and paired normal adjacent tissues by RT-qPCR, Western blotting and immunohistochemical staining. The data demonstrated that the expression of Rictor is significantly increased in the NSCLC tissues as compared with the adjacent tissues at both mRNA and protein levels** (Figure 1C-E, Supplementary Table 3)**. These results indicate that Rictor might be a critical molecule in NSCLC development. Moreover, the observation that Rictor protein levels correspond exactly with its mRNA levels suggests that Rictor expression is regulated, at least in part, at the transcriptional level in human NSCLC.

### Rictor is closely related to the tumorigenic properties of NSCLC cells

**Figure 2:**
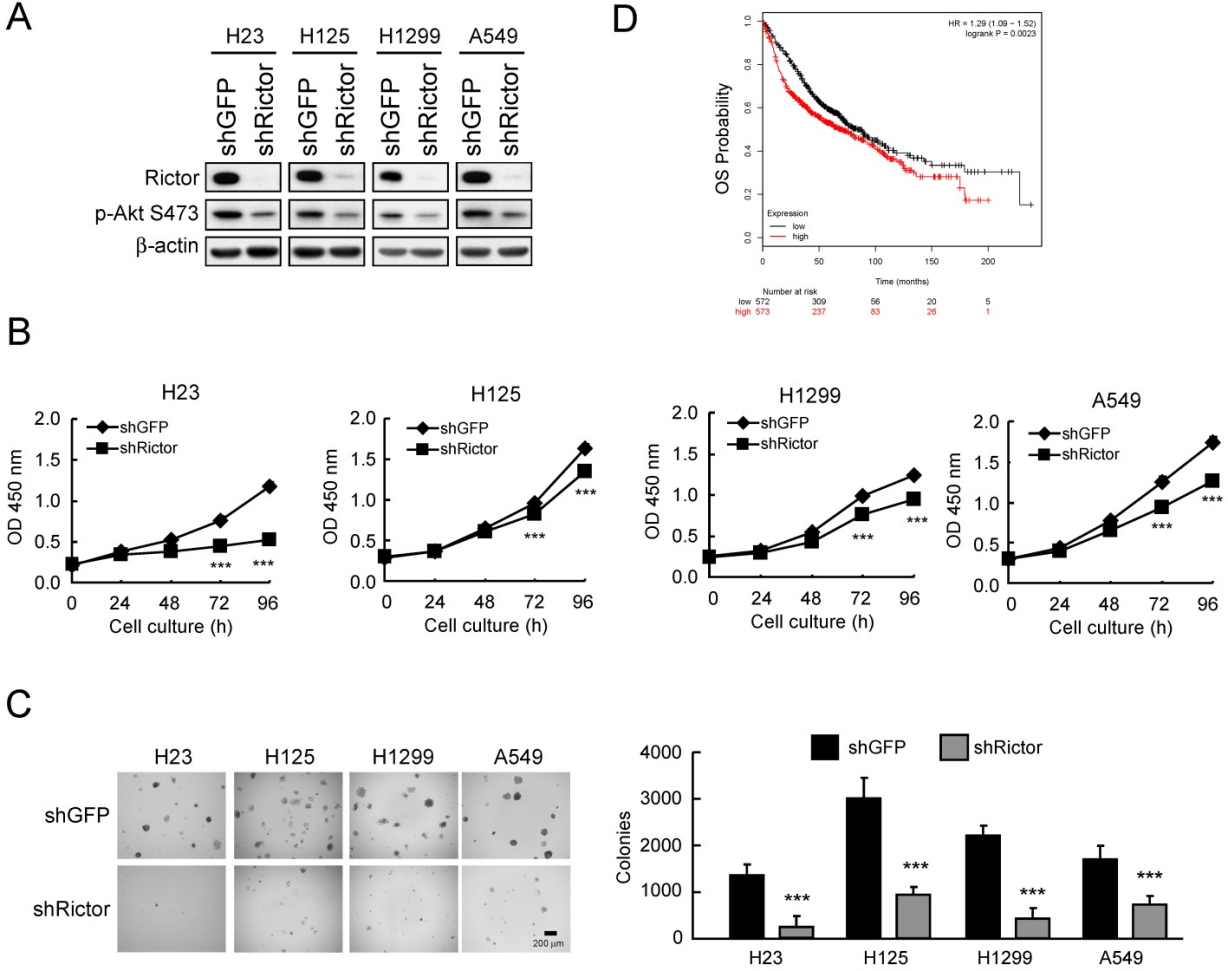
Rictor correlates with the tumorigenic properties of NSCLC cells.** A**, knockdown of Rictor in NCSLC cell lines and cell lysates were analyzed by Western blotting. β-actin was as a loading control. **B**, knockdown of Rictor inhibited the proliferation of NSCLC cells. WST-1 assays were performed. Data represent mean ± SD from three independent experiments. ***p<0.001, significant difference compared with the shGFP cells. **C**, knockdown of Rictor attenuated anchorage-independent growth of NSCLC cells. Soft agar assays were performed. Data represent mean ± SD from two independent experiments. ***p<0.001, significant difference compared with the shGFP cells. **D**, Kaplan-Meier survival analysis for the relationship between survival time and Rictor signature in lung cancer was performed using the online tool (http://kmplot.com/analysis/). OS, Overall Survival. *p*<0.05 was considered to be a statistically significant difference.

Based on the observation that Rictor is highly expressed in NSCLC cell lines and tissues**,** we hypothesized that Rictor might affect the tumorigenic properties in NSCLC. Thus, we generated Rictor knockdown stable NSCLC cell lines using lentivirus-mediated RNA interference and assessed the tumorigenic properties of NSCLC cells. The results showed that in H23, H125, H1299 and A549 cells with Rictor knockdown, the phosphorylation level of Akt Ser473, a substrate of Rictor-containing mTORC2 kinase complex 35, is consistently reduced by Rictor hairpin** (Figure 2A)**. Knockdown of Rictor suppressed proliferation **(Figure 2B)** and anchorage-independent growth **(Figure 2C)** of these NSCLC cell lines. Rictor overexpression was positively correlated with lymph node metastasis (*p* = 0.0109, **Supplementary Table 4**). To further extend our observations to a clinicopathologically relevant context, we performed a Kaplan-Meier survival analysis of Rictor with an online tool (http://kmplot.com/analysis/). The results showed that higher Rictor expression was associated with worse overall survival (OS, *p* = 0.0023) **(Figure 2D)**. These results suggest that Rictor plays an important role in the tumorigenic and metastatic properties of NSCLC cells as well as Rictor overexpression correlates with poor prognosis in patients with NSCLC.

### Rictor regulates migration, invasion and EMT reprogramming in NSCLC cells

**Figure 3:**
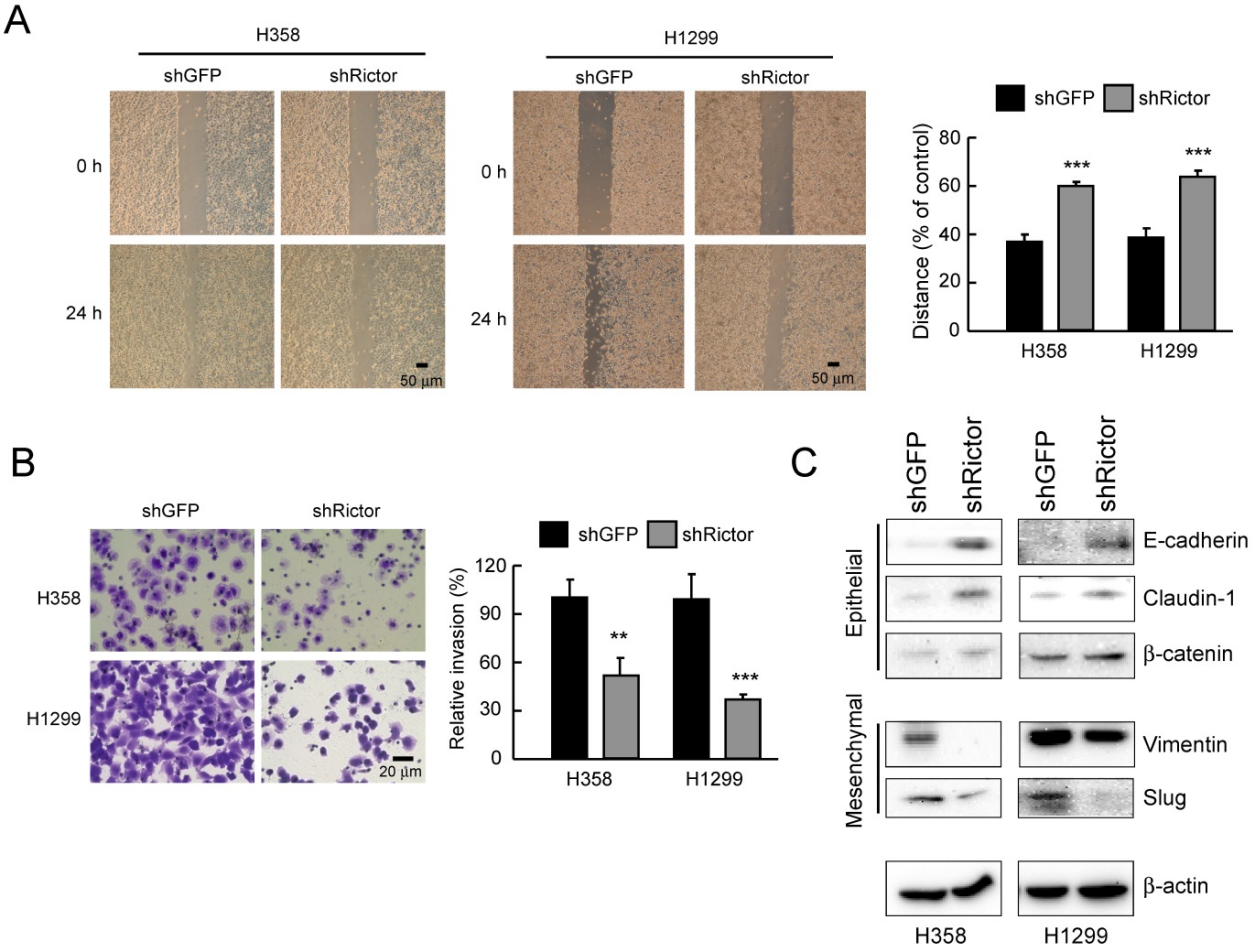
Rictor involves in the regulation of migratory and invasive abilities of NSCLC cells.** A**, knockdown of Rictor attenuated the migratory ability of H358 and H1299 NSCLC cells. Stable Rictor knocking down H358 and H1299 cells were subjected to wound healing assays. Images were taken at 0, 24 h (*left panel*). Knockdown of Rictor on the migratory ability of H358 and H1299 cells was evaluated by measuring the width of the wound area at each time point (*right panel*). Data represent mean ± SD from three fields per treatment for three independent experiments. ***p<0.001, significant difference compared with the shGFP cells.** B**, knockdown of Rictor attenuated the invasive ability of H358 and H1299 NSCLC cells. Stable Rictor knocking down H358 and H1299 cells were subjected to transwell invasion assays. The invaded cells were then photographed under a light microscope (*left panel*) and quantified (*right panel*). Data represent mean ± SD from three fields per treatment for two independent experiments. **p<0.01, ***p<0.001, significant difference compared with the shGFP cells. **C**, stable knockdown of Rictor in H358 and H1299 cells and the level of mesenchymal and epithelial markers were examined by Western blotting with specific antibodies. β-actin was as a loading control.

To elucidate the role of Rictor in NSCLC progress, NSCLC cells were silenced Rictor expression by RNA interference. Migratory capacity was assessed by wound healing assays, while the invasive potential was analyzed using Matrigel-coated transwell chambers. The result indicated that knockdown of Rictor significantly decreased the migratory capabilities of both H358 and H1299 cells** (Figure 3A)**. Also, suppression of Rictor decreased the invasive abilities of both H358 and H1299 cells **(Figure 3B)**. We next wanted to identify how Rictor regulates the migratory and invasive phenotypes of NSCLC cells. EMT is a critical step in metastasis involving different biological mechanisms 41. The expression of EMT markers and EMT-related transcription factors were then investigated. The results showed that knockdown of Rictor in both H358 and H1299 cells decreased the expression of mesenchymal marker vimentin and the E-cadherin transcriptional repressor Slug 41** (Figure 3C)**. On the contrary, expression of the epithelial markers E-cadherin and β-catenin, which is required for the coupling of E-cadherin to the actin cytoskeleton 23, 42, were increased upon knockdown of Rictor. The epithelial marker and tight junction protein claudin-1 was also significantly upregulated **(Figure 3C)**. These results suggested that the inhibition of Rictor resulted in MET, which is a reverse process of EMT. These date demonstrate that Rictor participates in the regulation of cell motility, invasiveness and EMT reprogramming in NSCLC cells.

### Transcription factor KLF4 regulates Rictor expression

**Figure 4:**
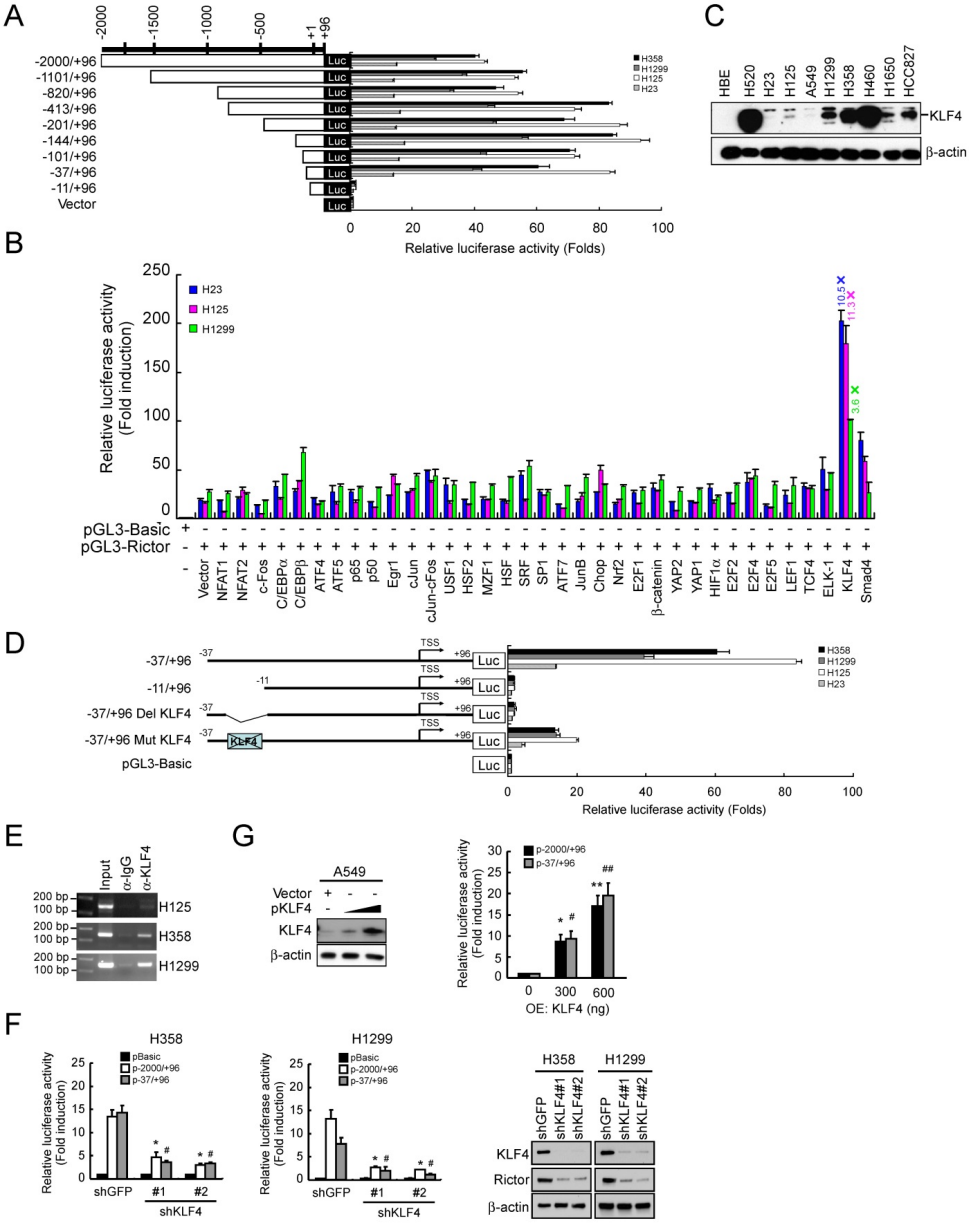
KLF4 regulates the transcription activity of the human *Rictor* promoter. **A**, H358, H1299, H125 and H23 cells were transfected with various human *Rictor* promoter constructs and subjected to dual-luciferase reporter assays. Luciferase activities were normalized to a co-expressed Renilla luminescent signal and were shown as relative folds against control samples. **B**, H23, H125 and H1299 cells were co-transfected a *pGL3-37/+96* with various transcription factor expression constructs and subjected to dual-luciferase reporter assays. Luciferase activities were normalized to a co-expressed Renilla luminescent signal and were shown as relative folds against control samples. **C**, expression of KLF4 level in NSCLC cell lines was examined by Western blot analysis with specific antibodies. β-actin was as a loading control. **D**, H358, H1299, H125 and H23 cells were transfected with the indicated constructs and subjected to dual-luciferase reporter assays. The positions of the putative KLF4 binding site identified with MatInspector in the *Rictor* promoter were indicated. Luciferase activities were normalized to a co-expressed Renilla luminescent signal and were shown as relative folds against control samples. **E**, KLF4 is directly bound to the promoter region of the *Rictor* gene. H125, H358 and H1299 cells were subjected to ChIP assays with a KLF4 antibody. The size of the PCR product is 151 bp.** F**, knockdown of KLF4 inhibited the *Rictor* promoter activity and protein expression. H358-shGFP, H358-shKLF4, H1299-shGFP and H1299-shKLF4 cells were transfected with* pGL3-2000/+96* or *pGL3-37/+96* construct and subjected to dual-luciferase reporter assays. Luciferase activities were normalized to a co-expressed Renilla luminescent signal and were shown as relative folds against control samples. *p<0.05, significant difference compared with the *pGL3-2000/+96*-transfected shGFP cells. #*p*<0.05, a significant difference compared with the *pGL3-37/+96-*transfected shGFP cells (*left and middle panels*). Effect of KLF4 knocking down on Rictor protein level in H358 and H1299 cells was examined by Western blotting with specific antibodies. β-actin was as a loading control (*right panel*). **G**, overexpression of KLF4 increased the *Rictor* promoter activity. A549 cells were co-transfected *pGL3-2000/+96* or *pGL3-37/+96* construct with a KLF4 expression construct and subjected to Western blot analysis (*left panel*) and dual-luciferase reporter assays (*right panel*). Luciferase activities were normalized to a co-expressed Renilla luminescent signal and were shown as relative folds against control samples. *p<0.05, **p<0.01, significant difference compared with the *pGL3-2000/+96* and* vector*-transfected A549 cells. #*p*<0.05, ##*p*<0.01, significant difference compared with the *pGL3-37/+96* and* vector-*transfected A549 cells.

Our data indicated that knockdown of Rictor resulted in switching from mesenchymal phenotype to epithelial phenotype. We next wanted to identify how Rictor itself is transcriptionally regulated. Constructs containing various lengths of the human *Rictor* promoter were cloned to drive the firefly luciferase gene expression and analyzed the reporter constructs by transient transfection of NSCLC cell lines. The dual reporter gene assay results indicated that the transcriptional activity of sequential truncations from -2000 to -37 of the *Rictor* promoter was not decreased. However, the transcriptional activity of *pGL3-11/+96* was dramatically decreased to the basal level in four NSCLC cell lines tested (**Figure 4A**). The results indicated that the *-37/-11* region is critical for the *Rictor* gene transcription. Based on this result, the putative transcription factor binding sites on this region were predicted by online prediction programs (http://www.genomatix.de/ and http://www.gene-regulation.com/) (**Supplementary Figure 2**). To identify transcription factor(s) that regulated the *Rictor* promoter activity, the *pGL3-37/+96* was cotransfected with various transcription factor expression plasmids into H23, H125 and H1299 cells. The results showed that overexpression of KLF4 elevated the *pGL3-37/+96* transcriptional activity by 10.5-fold in H23 cells, 11.3-fold in H125 cells and 3.6-fold in H1299 cells (**Figure 4B**), indicating that KLF4 contributes to the expression of Rictor. In addition, the magnitude of the induction of *pGL3-37/+96* transcriptional activity by exogenous KLF4 was negatively correlated with the basal level of endogenous KLF4 in H23, H125 and H1299 cells **(Figure 4C)**. Based on the predicted binding site of KLF4 (**Supplementary Figure 2**), KLF4 binding site deletion mutant (*pGL3-37/+96 Del KLF4*) and site-directed mutant (*pGL3-37/+96 Mut KLF4*) were constructed from the wild-type construct *pGL3-37/+96*, as indicated in** Figure 4D**. The constructs were respectively transfected into four NSCLC cell lines. The results showed that the transcriptional activity of *pGL3-37/+96 Del KLF4* was decreased to the basal level and the transcriptional activity of* pGL3-37/+96 Mut KLF4* was dramatically decreased compared with its wild-type counterpart in four NSCLC cell lines tested (**Figure 4D**). Moreover, ChIP results indicated that KLF4 is directly bound to the *Rictor* promoter (**Figure 4E**). Knockdown of KLF4 by RNA interference parallels the reduction in Rictor protein level and the luciferase expression driven by the *Rictor* promoter in H358 and H1299 cells (**Figure 4F**), indicating that KLF4 regulated the expression of* Rictor* gene in these NSCLC cells. To further determine whether KLF4 directly activates the *Rictor* promoter, the *pGL3-2000/+96* or* pGL3-37/+96* reporter was co-transfected with KLF4 full-length expression plasmid into A549 cells whose basal KLF4 protein level was extremely low **(Figure 4C)**. The results demonstrated that overexpression of KLF4 elevated the *Rictor* promoter activity dose-dependently in A549 cells **(Figure 4G)** as well as Rictor protein level in A549 and H23 cells **(Supplementary Figure 3)**. Collectively, the data suggest that transcription factor KLF4 directly binds to the *Rictor* promoter and regulates Rictor expression in NSCLC cells.

### Knockdown of KLF4 prevents Rictor-mediated EMT, mTOR/Rictor interaction, NSCLC migration and invasion

**Figure 5:**
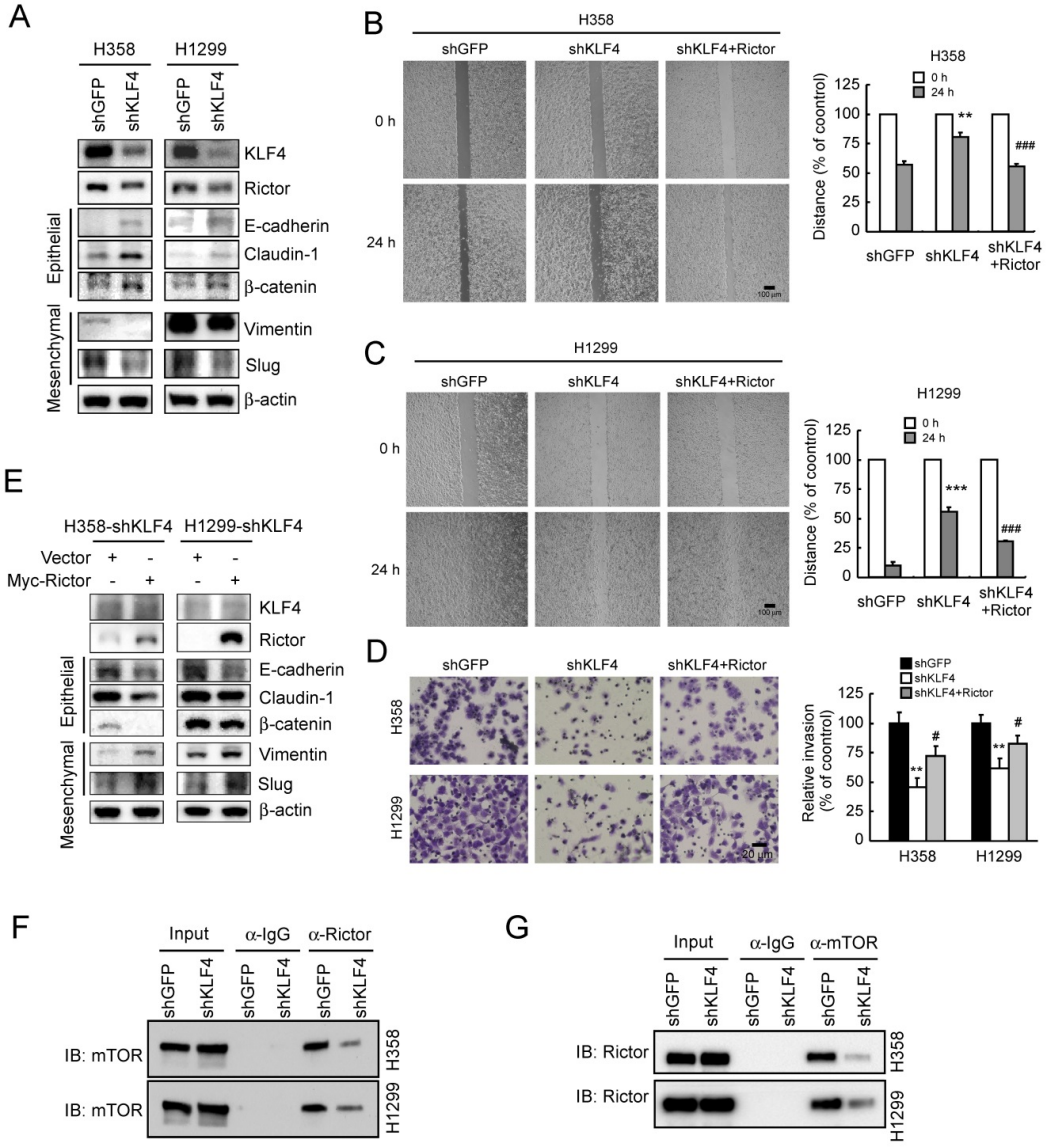
Knockdown of KLF4 prevents Rictor-mediated EMT, mTOR/Rictor interaction and NSCLC cell migration and invasion.** A**, knockdown of KLF4 in H358 and H1299 cells and the levels of Rictor, mesenchymal and epithelial markers were examined by Western blotting with specific antibodies. β-actin was as a loading control. **B and C**, re-introduction of Rictor rescues the inhibition of cell migration caused by KLF4 knocking down. Stable H358-shKLF4** (B)** and H1299-shKLF4 **(C)** cells were transfected with a Rictor expression construct and subjected to a wound healing assay. **p<0.01, ***p<0.001, significant difference compared with the shGFP cells. ###*p*<0.001, a significant difference compared with the shKLF4 cells.** D**, re-introduction of Rictor attenuates the inhibition of cell invasion caused by KLF4 knocking down. Stable H358-shKLF4 and H1299-shKLF4 cells were transfected with a Rictor expression construct and subjected to transwell invasion assays. **p<0.01, a significant difference compared with the shGFP cells. #*p*<0.05, a significant difference compared with the shKLF4 cells. **E**, re-introduction of Rictor affects a series of characteristic changes of MET. Stable H358-shKLF4 and H1299-shKLF4 cells were transfected with a Rictor expression construct and KLF4, Rictor, mesenchymal and epithelial markers were examined by Western blotting with specific antibodies. β-actin was as a loading control. **F and G**, knockdown of KLF4 impedes mTOR/Rictor interaction in NSCLC cells. H358-shGFP, H358-shKLF4, H1299-shGFP and H1299-shKLF4 cells were lysed and immunoprecipitated with a Rictor, mTOR, or normal IgG antibody. The immune complexes and input were analyzed by immunoblotting with an mTOR** (F)** or Rictor **(G)** antibody.

Since Rictor is involved in EMT regulation in NSCLC cells **(Figure 3)**, to explore the role of KLF4 in Rictor-mediated EMT, we suppressed KLF4 expression by RNA interference in H1299 and H358 cells. As shown in **Figure 5A**, knockdown of KLF4 resulted in Rictor's downregulation accompanied by a decrease in vimentin and Slug expression. However, the epithelial markers E-cadherin, claudin-1 and β-catenin were upregulated upon KLF4 knocking down. A phenotypic hallmark of EMT is stimulation of cell migration and invasion. Therefore, we assessed NSCLC cell migration by wound healing assays. Compared with the control cells, knockdown of KLF4 significantly reduced the observed increase in migration (**Figure 5B and 5C**) and invasion (**Figure 5D**) of both H358 and H1299 cells. To further confirm the role of KLF4 in Rictor-regulated EMT, we re-introduced Rictor in stable H358-shKLF4 and H1299-shKLF4 cells. The results indicated that overexpression of Rictor partially compromised the inhibition of cell migration (**Figure 5B and 5C**) and invasion **(Figure 5D)** caused by KLF4 knocking down. Moreover, the mesenchymal and epithelial markers were also partially reversed by overexpression of Rictor in both H358-shKLF4 and H1299-shKLF4 cells **(Figure 5E)**. We also reintroduced KLF4 in KLF4 knockdown stable H1299 cells to exclude the potential off-target effect of KLF4 knocking down and performed wound healing assays and transwell invasion assays. The results indicated that re-expression of KLF4 partially compromised the inhibition of cell migration and invasion by KLF4 knocking down (**Supplementary Figure 4**). Rictor has been reported to interact with mTOR 21, ILK 9, 24, PKC ζ 10, myosin Myo1c 8 or Cullin-1 11 to form a complex. Among them, Rictor/mTOR 12, 19, 21 and Rictor/ILK 24 have been involved in EMT reprogramming. We therefore sought to elucidate whether KLF4 affects Rictor/mTOR or Rictor/ILK interaction and finally regulates Rictor-mediated EMT in NSCLC cells. We immunoprecipitated endogenous Rictor in H358 and H1299 cells and found the presence of mTOR in Rictor immunoprecipitates **(Figure 5F)**. Reciprocal immunoprecipitation experiments were also done using H358 and H1299 cells, confirming the presence of Rictor in mTOR immunoprecipitates **(Figure 5G)**, indicating that knockdown of KLF4 diminished the Rictor/mTOR interaction. However, we did not detect the Rictor/ILK interaction** (data not shown)**, suggesting that Rictor did not form complex with ILK to regulate Rictor-mediated EMT in NSCLC cells. In summary, these data indicate that KLF4 involves in Rictor expression and Rictor-mediated EMT reprogramming.

### Knockdown of KLF4 prevents tumor metastasis of NSCLC* in vivo*

**Figure 6:**
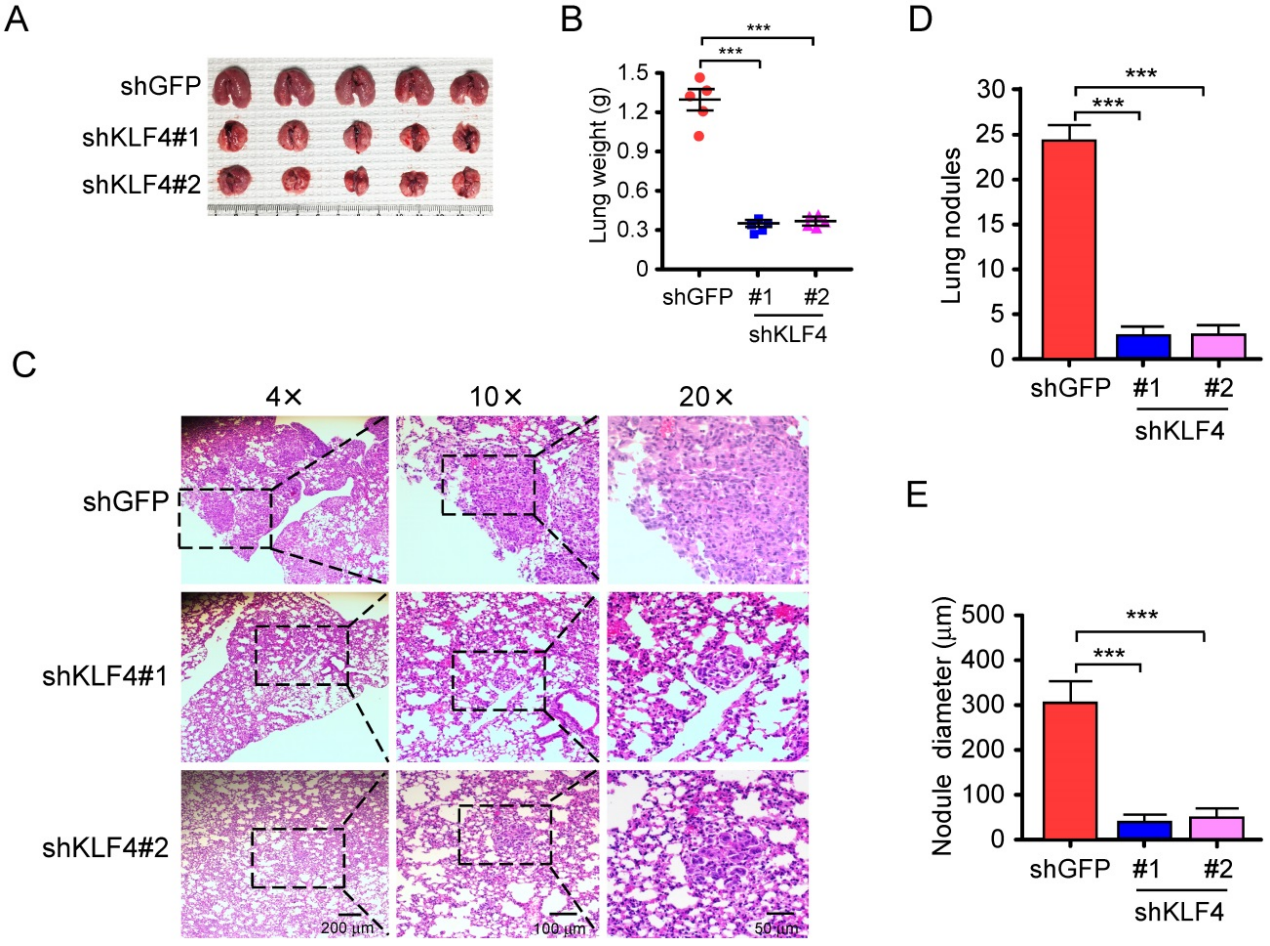
Knockdown of KLF4 suppresses tumor metastasis of NSCLC* in vivo*.** A and B**, H1299-shGFP and H1299-shKLF4 cells were injected into the lateral tail vein of nude mice as described in Materials and methods. Tumor mass was photographed** (A)**, and weight** (B)**. ***p<0.001, significant difference compared with the shGFP group.** C**, H&E staining was applied to verify the metastatic nodules in H1299-shGFP and H1299-shKLF4 groups. **D**, the average tumor number of lung micro-metastasis per mouse from each group was determined. ***p<0.001, significant difference compared with the shGFP group. **E**, the average tumor size of lung micro-metastasis per mouse from each group was determined. ***p<0.001, significant difference compared with the shGFP group.

Migratory and invasive capacities are essential for the cancer cells in the primary sites and the extravasated cancer cells in distant organs to successfully establish final metastasis 43. Therefore, *in vivo* metastasis assay was carried out to assess the effect of KLF4 knocking down on metastasis. H1299-shGFP and H1299-shKLF4 cells were injected through the tail vein of nude mice. After 6 weeks, a larger size and greater weight of lungs were found in the shGFP group due to the formation of lung tumors **(Figure 6A and 6B)**. The lung tissues were further examined with hematoxylin and eosin (HE) staining for lung metastasis **(Figure 6C)**. The results indicated that the number **(Figure 6D)** and size** (Figure 6E)** of metastatic nodules in the lung were dramatically decreased in shKLF4 groups. The results indicate that knockdown of KLF4 prevents tumor metastasis of NSCLC* in vivo.*

## Discussion

Although Rictor is frequently increased in various human tumors and exerts different functions in an mTOR-dependent or -independent manner leading to tumor growth and increased invasive characteristics, the mechanisms regulating Rictor expression remains poorly understood. Reports have described Rictor expression via post-translational control mechanism. For instance, FBXW7 interacts with Rictor and mediates its degradation, and this process requires phosphorylation of Rictor at Thr1695 by GSK3 44. In BRCA1-deficient triple-negative breast cancer (TNBC) cells, PARP3 inhibition exacerbates centrosome amplification and genome instability via limitation of Rictor protein level by ubiquitination, thus efficiently represses oncogenic Rictor/mTORC2 signaling 45. Additionally, Rictor is post-transcriptionally controlled by HuR binding to the 3′ UTR of the *Rictor* transcript, which led to enhancement of *Rictor* mRNA translation and elevated mTORC2 activity 46. Rictor is reportedly upregulated by FoxO1 at both mRNA and protein levels. However, the FoxO1-elevated Rictor expression appears to be independent of the DNA-binding activity of FoxO1, because the FoxO1 H215R mutant, which is impaired in DNA binding, was still able to elevate *Rictor* mRNA, suggesting that FoxO1 elevates Rictor expression through the association with another transcription factor that possesses DNA-binding activity 47. Therefore, the fine transcriptional control mechanism of Rictor expression needs to be explored.

Among the complexes of Rictor/mTOR, Rictor/ILK, Rictor/PKC ζ, Rictor/Myo1c and Rictor/Cullin-1, the Rictor/mTOR and Rictor/ILK have been shown to involve in EMT reprogramming. It has been reported that Rictor/mTOR is involved in regulating EMT, motility, and metastasis of colorectal cancer via RhoA and Rac1 signaling pathways 21. Lamouille *et al*.^48^ reported that TGFβ induces mTORC2 kinase activity and enhances Rictor/mTOR formation during EMT. Knockdown of Rictor impairs TGFβ-induced downregulation of E-cadherin protein, compromises TGFβ-induced increase in N-cadherin and Snail expression in NMuMG cells, blocks TGFβ-induced EMT and invasive behavior of both NMuMG cells and E4 squamous carcinoma cells. The findings indicate that Rictor/mTOR-containing mTORC2 is a key driver of EMT. Prakash *et al.* 49 showed that in NMuMG cells, TGFβ-driven EMT induces rRNA-dependent association of Rictor with nucleoli. The finding that the EMT-associated ribosome biogenesis program directs Rictor's association with newly generated ribosomes aligns with the notion that mTORC2 activation drives EMT through physical interactions with the ribosome. ILK/Rictor complex is reportedly formed constitutively in breast cancer cells but not in normal epithelial cells. However, this complex can be induced in normal mammary epithelial cells after TGFβ treatment. Disruption of ILK/Rictor formation by silencing ILK or Rictor expression inhibits TGFβ-induced mesenchymal marker α-SMA but increases the epithelial marker β-catenin localized at adherens junctions, indicating that ILK/Rictor interaction is necessary for TGFβ-induced EMT. Furthermore, inhibition of ILK/Rictor formation promotes MDA-MB-231 breast cancer cells undergoing the reverse mesenchymal-epithelial transition, inducing MDA-MB-231cells from an aggressive mesenchymal phenotype to a normalized epithelial phenotype 24. Our results show that, though ILK/Rictor interaction is absent, mTOR/Rictor interaction plays a critical role in EMT reprogramming in NSCLC. Suppression of either Rictor itself or its upstream regulator KLF4 can reverse the mesenchymal phenotype to the epithelial phenotype of NSCLC cells. Our findings imply that both the Rictor-containing complex and its upstream regulator could act as responsive targets to inhibit EMT and prevent NSCLC cell invasion and metastasis.

As a member of Sp/KLF family, KLF4, which comprises both Sp1-like and KLF-like factors that bind to common Sp/KLF sites, including GC-boxes, GT-boxes and basic transcription elements, regulates the expression of a large number of target genes involved in a wide range of cellular functions, such as differentiation, proliferation and apoptosis 25, 50, 51. Although Sp/KLF members appear to bind with varying affinities to different DNA sequences, all family members have the potential to affect Sp/KLF site-dependent transcription in cells where they are expressed. Additionally, individual Sp/KLF family members, such as Sp1, Sp3, KLF4 and KLF5, can activate or repress transcription, rely on the cell line or promoter examined 31, 50, 52. For example, evidence has shown that KLF4 competes with Sp1 in promoter binding and suppresses the expression of cyclin D1 53. Sp3 and Sp4 can repress transcription by competing with Sp1 for the core cis-elements on the human *ADH5/FDH* minimal promoter 52. KLF5 and KLF4 have opposite effects on *SM22α* gene expression 54. Similarly, KLF4 and Sp1 can bind the same *RYBP* promoter to induce and suppress *RYBP* transcription, respectively 31. Our results show that in A549 and H23 cells, where KLF4 expression is barely detectable **(Figure 4C)**, Rictor is still abundantly expressed, suggesting that in addition to KLF4, other Sp/KLF family members or other transcription factors might contribute to Rictor regulation.

The functions of KLF4 are diverse and cell-type specific. KLF4 reportedly acts as a tumor suppressor in gastric cancer 55, colorectal cancer 56, bladder cancer 57 and prostate cancer 58, where a significant decrease or loss in KLF4 expression is frequently observed. Re-induced KLF4 expression in KLF4-null PC3 cells inhibits PC3 growth in monolayer and soft agar cultures 59. Conversely, KLF4 may also function as an oncogene, as it is overexpressed in human breast cancer 60, 61, oropharyngeal epidermoid carcinoma 62, skin squamous cell carcinomas 63, head and neck squamous cell carcinoma (HNSCC) 62, 64, related to an aggressive course 65. HNSCC patients with persistent KLF4 expression independently correlated with worse disease-specific survival. Enforced KLF4 expression in HNSCC cells increased* in vitro* migration and invasion abilities as well as *in vivo* tumorigenicity, illustrating that persistent KLF4 expression predicts poor prognosis and confers aggressiveness in HNSCC 66. As for lung cancer, KLF4 protein is reportedly downregulated in primary lung tumors compared with matched normal lung tissues, and enforced expression of KLF4 results in marked inhibition of cell growth and clonogenic formation, indicating that KLF4 acts as a tumor suppressor 67. Nevertheless, KLF4 is observably overexpressed in c-Met-overexpressing NSCLC cells and tissues. Knockdown of KLF4 reduces tumorigenic properties and increases gefitinib sensitivity in gefitinib-resistant NSCLC cells with c-Met overexpression, suggesting that KLF4 is an oncogene and contributes to gefitinib resistance in c-Met amplification-mediated gefitinib-resistant NSCLC cells 68. Naranjo Gómez *et al*. 69 showed that high expression of KLF4 in large-cell neuroendocrine lung carcinomas and small-cell lung cancers (SCLCs), which represents the fast-growing nature of this type of lung cancer that is considered highly lethal. Another study showed a significant decrease in KLF4 expression in adenocarcinomas compared with that in normal tissue, while significant KLF4 overexpression was detected in SCLCs. Furthermore, KLF4 was significantly increased in tumor stages II, III and IV compared with stage I in adenocarcinomas tissues, suggesting that the increase in KLF4 expression may associate with decreased tumor differentiation, increased aggressiveness and carcinogenic process 70. Our results showed that, though it appears no statistically significant difference, an upward trend of KLF4 expression in NSCLC tissues compared with matched normal lung tissues and a trend of KLF4 and Rictor positive correlation were observed (**Supplementary Figure 5A and 5B**), suggesting that KLF4 expression level was upregulated in our NSCLC cohort. The WB result also demonstrated that KLF4 was upregulated to varying degrees in NSCLC cell lines compared with the normal cell line HBE (**Figure 4C**). Importantly, increased KLF4 expression was associated with a worse OS among lung cancer patients from an online database (**Supplementary Figure 5C**). Our results present here indicate that KLF4 positively regulates Rictor-mediated epithelial-mesenchymal transition as well as tumor metastasis of NSCLC* in vivo* and may function as a tumor-promoting gene, which is partially consistent with those of Fadous-Khalifé *et al*. 70, Feng* et al*. 68 and Naranjo Gómez *et al*. 69 but not consistent with those of Hu *et al*. 67. The discrepancy may be due to the different histological subtypes in the sample cohorts, the sample numbers collected for study, and the different techniques for evaluating KLF4 expression. Thus, simple dichotomization between tumor suppressor gene and oncogene may not be appropriate for KLF4 in NSCLC. Interestingly, Ting *et al.* 71 recently reported that compared with primary tumor cells, the level of KLF4 was elevated in circulating tumor cells (CTCs), suggesting KLF4 expression contributes to cancer cell colonization and metastasis. The mechanisms underlying the variable KLF4 expression levels in NSCLC and how KLF4 functions in the development and progression of lung cancer, including NSCLCs, are worthy of future investigations.

In summary, we identify, for the first time, the human *Rictor* core promoter and confirm that transcription factor KLF4 directly binds to the *Rictor* promoter and transcriptionally upregulated Rictor expression. Knockdown of KLF4 results in Rictor's downregulation accompanied by a series of characteristic changes of MET and significantly decreases migration, invasion and metastasis of NSCLC cells. Re-introduction of Rictor in KLF4-knockdown NSCLC cells partially reverses the epithelial phenotype to the mesenchymal phenotype and attenuates the inhibition of cell migration and invasion caused by KLF4 knocking down. Knockdown of KLF4 prevents mTOR/Rictor interaction and tumor metastasis of NSCLC* in vivo*. Beyond the Rictor-targeted strategy, our discovery reveals that the suppression of the upstream regulator of Rictor may be a compelling therapeutic approach that deserves further study for the prevention of cancer metastasis.

## Supplementary Material

Supplementary figures and tables.
