# Supplementary material for: Epithelial-mesenchymal reprogramming by KLF4-regulated Rictor expression contributes to metastasis of non-small cell lung cancer cells
Source: Int J Biol Sci. 2022 Jul 18;18(13):4869–83. doi: 10.7150/ijbs.73548 (PMC9379402; doi:10.7150/ijbs.73548)
Supplement: Supplementary file 1 — Supplementary figures and tables. [file ijbsv18p4869s1.pdf]

Supplementary Figure 1

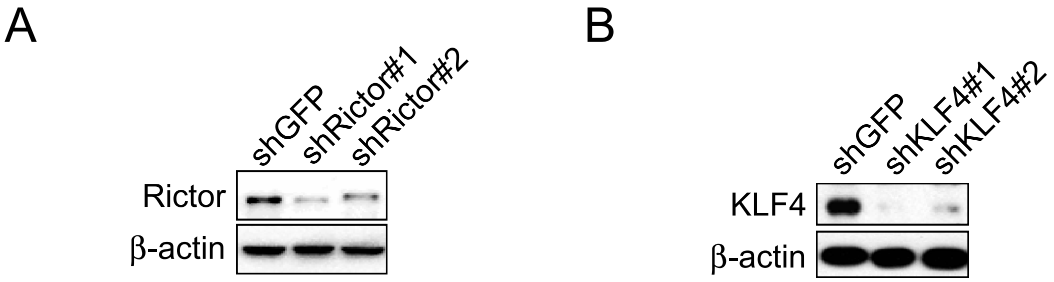

Supplementary Figure 2

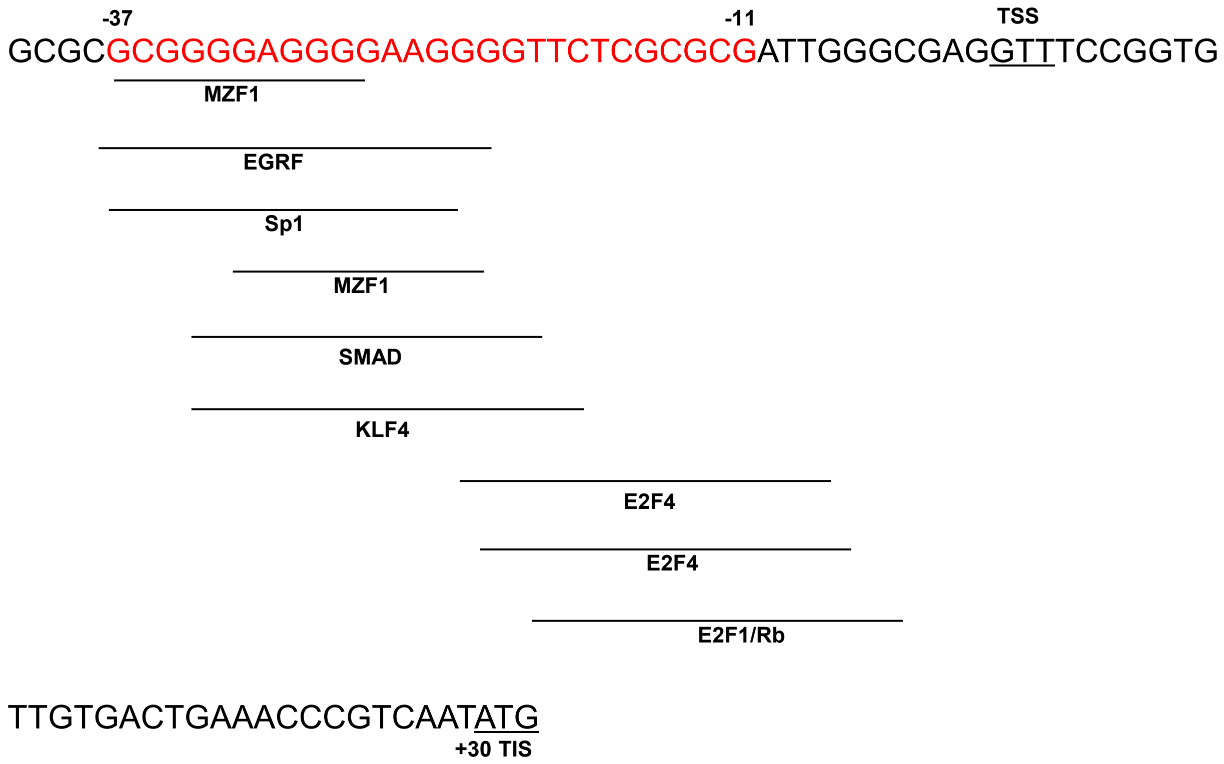

Supplementary Figure 3

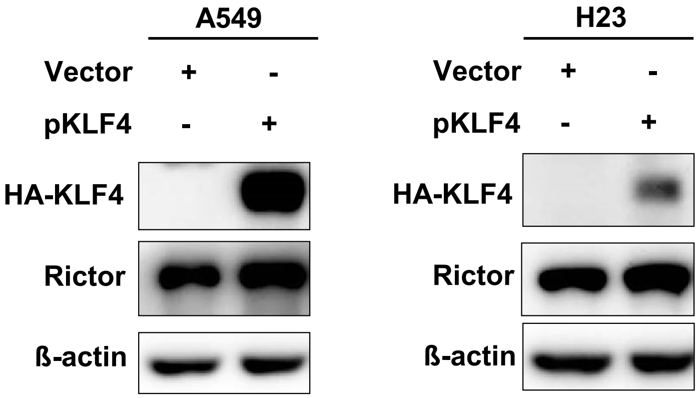

Supplementary Figure 4

A

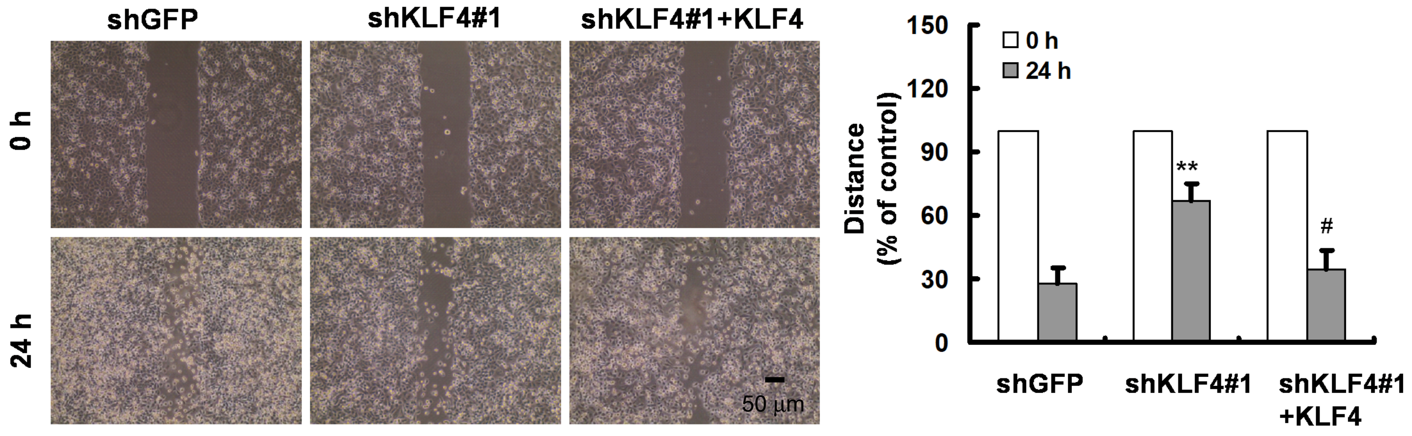

B

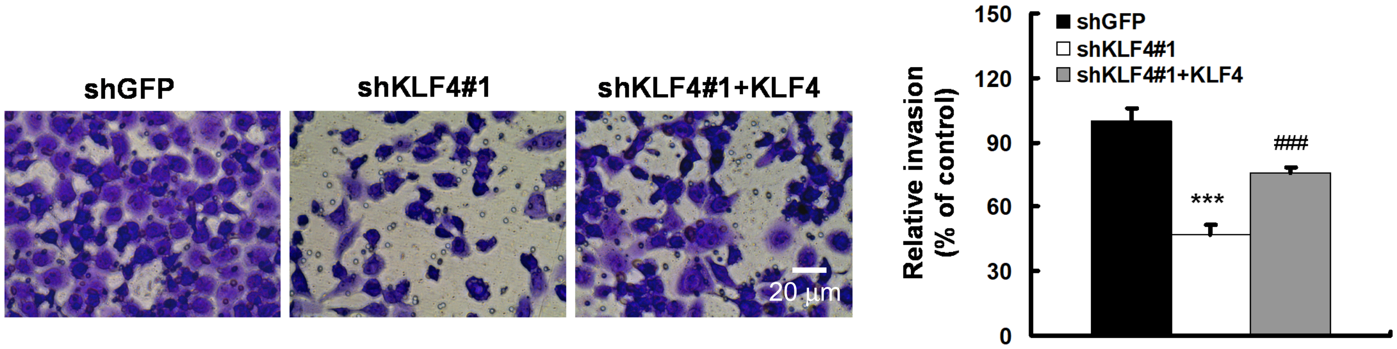

Supplementary Figure 5

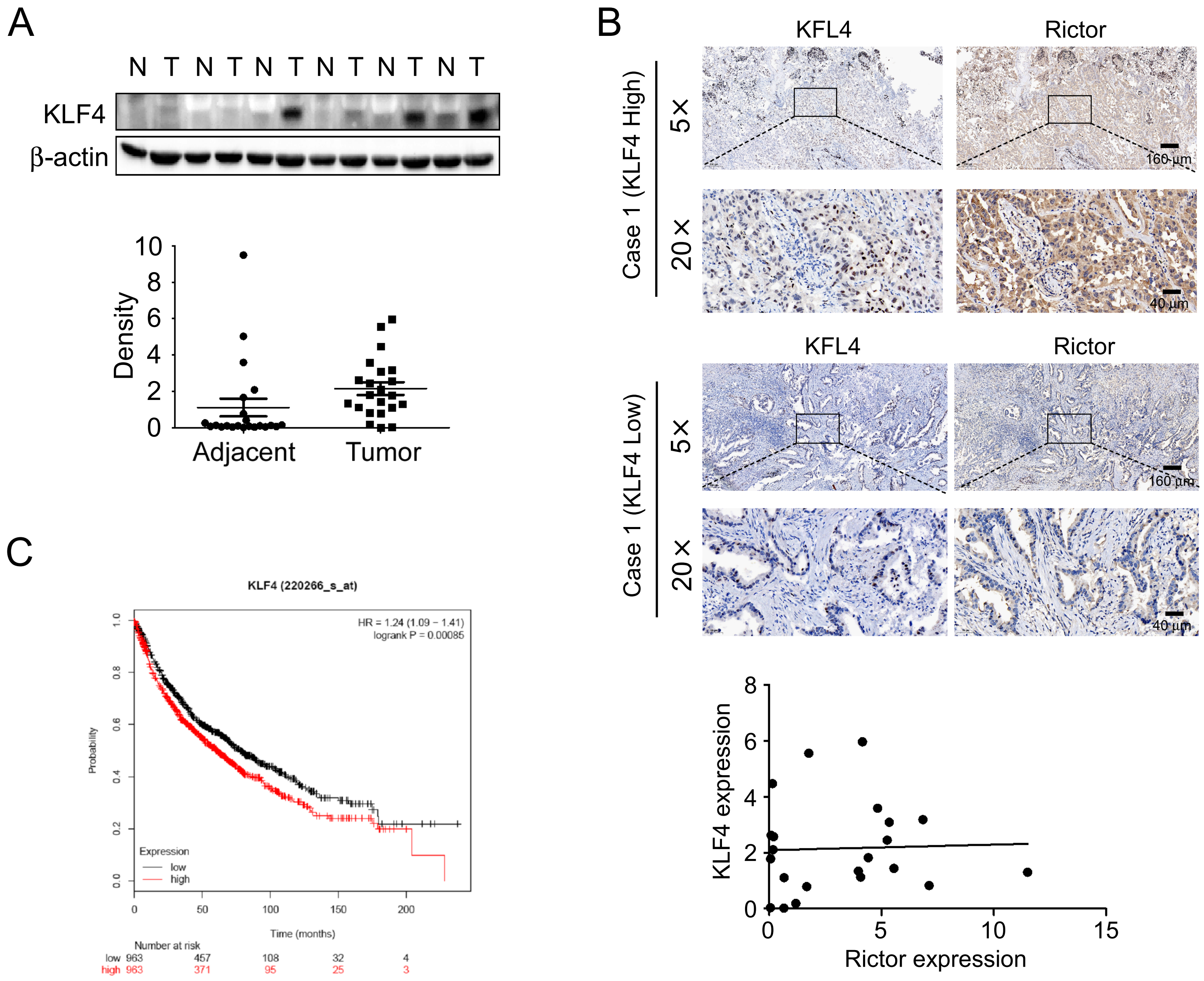

Supplementary Table 1. Primers for cloning of the constructs of human *Rictor* promoter

Gene region	Sense sequence (5'to3')	Antisense sequence (5'to3')
-2000/+96	ccgctcgagaagctataacggagggagcac	cccaagcttgttgcagcgggcttacct
-1101/+96	ccgctcgagagctgggaaaacagaactgg	cccaagcttgttgcagcgggcttacct
-820/+96	ccgctcgagaatccacaacacctgcatga	cccaagcttgttgcagcgggcttacct
-413/+96	ccgctcgagtctatggcagggcttcagag	cccaagcttgttgcagcgggcttacct
-201/+96	ccgctcgaggcggacgtgccctccgggtc	cccaagcttgttgcagcgggcttacct
-144/+96	ccgctcgagccttgggtcggctcagtg	cccaagcttgttgcagcgggcttacct
-101/+96	ccgctcgaggcaccacccgccgccgtcgc	cccaagcttgttgcagcgggcttacct
-37/+96	ccgctcgaggcggggaggggaaggggttc	cccaagcttgttgcagcgggcttacct
-11/+96	ccgctcgaggcgattgggcgaggtttccg	cccaagcttgttgcagcgggcttacct

Supplementary Table 2. Two-step PCR primers for cloning of the mutant *Rictor* promoter

Gene region	Sense sequence (5'to3')	Antisense sequence (5'to3')
Del KLF4	gctagcccgggctcgaggcgcgattgggcgagg	cggaaacctcgcccaatcgcgcctcgagcccgggctagc
Mut KLF4	gcggggaggtgtaggggttctcgcgcgattg	gaacccctacacctccccgcctcgag

Supplementary Table 3. Protein expression of Rictor in NSCLC tissues and adjacent non-tumor tissues.

Tissue sample	No. of patients	Rictor		*p*- value
		Low	High	
Tumor	22	6	16	<0.0001*
Adjacent	22	20	2	

Chi-square test.

*$p<0.05$ indicates a significant association among the variables.

Supplementary Table 4. Relationships between the expression of Rictor and clinicalpathological characteristics in 22 patients with NSCLC

p–value* Characteristics	All cases	Rictor Non-overexpression	Rictor Over-expression	
**Gender**				0.4805
Male	20	5	15	
Female	2	1	1	
**Age (years)**				0.3476
≤60	10	4	6	
>60	12	2	10	
**Differentiation grade**				1.0000
Well, moderately	18	5	13	
Poorly	4	1	3	
**Initial clinical stage**				1.0000
≤ II a	7	2	5	
> II a	15	4	11	
**Tumor stages**				1.0000
T1 + T2	3	1	2	
T3 + T4	19	5	14	
**Nodal metastasis**				**0.0109**
Negative	8	5	3	
Positive	14	1	13	

**p*-value was calculated using χ^2 test or Fisher's exact test. $p<0.05$ indicates a significant association among the variables.

Supplementary Figure legends

Supplementary Figure 1. Stable knockdown of Rictor or KLF4 in H1299 NSCLC cells. **A**, H1299 cells were stably transfected with a GFP shRNA or Rictor shRNAs. Cell lysates were harvested and knockdown of Rictor was confirmed by Western blot analysis. **B**, H1299 cells were stably transfected with a GFP shRNA or KLF4 shRNAs. Cell lysates were harvested and knockdown of KLF4 was confirmed by Western blot analysis.

Supplementary Figure 2. Prediction of the putative transcription factor binding sites of human *Rictor* promoter. TSS: Transcription start site. TIS: Translation initiation site.

Supplementary Figure 3. Overexpression of KLF4 increased the Rictor protein level. A549 and H23 cells were transfected with a *KLF4* expression construct and expression of KLF4 and Rictor was assessed by Western blot analysis with an anti-HA antibody and an anti-Rictor antibody, respectively.

Supplementary Figure 4. Re-introduction of KLF4 rescues the inhibition of cell migration and invasion caused by KLF4 knocking down. **A,** stable KLF4 knockdown H1299 cells were reintroduced KLF4 and subjected to wound healing assays. **p<0.01, significant difference compared with the shGFP cells. #p<0.05, a significant difference compared with the shKLF4 cells. **B,** stable KLF4 knockdown H1299 cells were reintroduced KLF4 and subjected to transwell invasion assays. ***p<0.001, a significant difference compared with the shGFP cells. ###p<0.001, a significant difference compared with the shKLF4 cells.

Supplementary Figure 5. Expressions of KLF4 in human non-small cell lung cancer. **A**, Western blot analysis was performed to examine KLF4 expression in six representative NSCLC cases. β-actin was as a loading control. N, normal adjacent tissue; T, tumor (*upper panel*). Western blotting determined KLF4 protein levels in the malignant and the corresponding normal adjacent tissues of 22 NSCLC patients (*lower panel*). The intensity was evaluated using Image J (NIH) computer software. p>0.05. **B**, representative images of immunohistochemical staining for KLF4 and Rictor in represent specimens among 22 cases of human NSCLC (*upper panel*). Scatterplot showed the correlation between the expression of KLF4 and Rictor in human NSCLC tissues of 22 NSCLC patients (*lower panel*). Pearson's coefficient tests were performed to assess statistical significance. p>0.05. **C**, Kaplan-Meier survival analysis for the relationship between survival time

and KLF4 signature in lung cancer was performed by using the online tool (http://kmplot.com/analysis/).

$p<0.05$ was considered to be a statistically significant difference.

Supplementary Table 1. Primers for cloning of the constructs of human *Rictor* promoter.

Supplementary Table 2. Two-step PCR primers for cloning of the mutant *Rictor* promoter.

Supplementary Table 3.Protein expression of Rictor in NSCLC tissues and adjacent non-tumor tissues.

Supplementary Table 4. Relationships between the expression of Rictor and clinicalpathological characteristics in 22 patients with NSCLC.
